# Improving obstetric care in low-resource settings: implementation of facility-based maternal death reviews in five pilot hospitals in Senegal

**DOI:** 10.1186/1478-4491-7-61

**Published:** 2009-07-23

**Authors:** Alexandre Dumont, Caroline Tourigny, Pierre Fournier

**Affiliations:** 1 UR10 « santé de la mère et de l'enfant en milieu tropical », Institut de Recherche pour le Développement, Dakar, Sénégal; 2 Unité de Santé Internationale, Centre de Recherche du Centre Hospitalier de l'Université de Montréal, Université de Montréal, Montréal, Canada

## Abstract

**Background:**

In sub-Saharan Africa, maternal and perinatal mortality and morbidity are major problems. Service availability and quality of care in health facilities are heterogeneous and most often inadequate. In resource-poor settings, the facility-based maternal death review or audit is one of the most promising strategies to improve health service performance. We aim to explore and describe health workers' perceptions of facility-based maternal death reviews and to identify barriers to and facilitators of the implementation of this approach in pilot health facilities of Senegal.

**Methods:**

This study was conducted in five reference hospitals in Senegal with different characteristics. Data were collected from focus group discussions, participant observations of audit meetings, audit documents and interviews with the staff of the maternity unit. Data were analysed by means of both quantitative and qualitative approaches.

**Results:**

Health professionals and service administrators were receptive and adhered relatively well to the process and the results of the audits, although some considered the situation destabilizing or even threatening. The main barriers to the implementation of maternal deaths reviews were: (1) bad quality of information in medical files; (2) non-participation of the head of department in the audit meetings; (3) lack of feedback to the staff who did not attend the audit meetings. The main facilitators were: (1) high level of professional qualifications or experience of the data collector; (2) involvement of the head of the maternity unit, acting as a moderator during the audit meetings; (3) participation of managers in the audit session to plan appropriate and realistic actions to prevent other maternal deaths.

**Conclusion:**

The identification of the barriers to and the facilitators of the implementation of maternal death reviews is an essential step for the future adaptation of this method in countries with few resources. We recommend for future implementation of this method a prior enhancement of the perinatal information system and initial training of the members of the audit committee – particularly the data collector and the head of the maternity unit. Local leadership is essential to promote, initiate and monitor the audit process in the health facilities.

## Background

In sub-Saharan Africa, maternal and perinatal mortality and morbidity are major problems for which progress has been inadequate. Reducing them is the aim of two of the Millenium Development Goals (MDG4 and MDG5); unfortunately, attainment of these goals in this part of the world is very unlikely [1]. The broad strategies that have made it possible to reduce maternal and perinatal mortality are known: prenatal care, management of labour and delivery by qualified personnel, and availability of emergency obstetric care (EmOC) [2]; however, their implementation is a major challenge in sub-Saharan Africa, where health care systems are fragile and often underdeveloped. Service availability and quality of care in health facilities are heterogeneous and most often inadequate [3-6].

In Senegal, the rate of maternal mortality estimated by the World Health Organization (WHO) in 2005 remained high: 980 maternal deaths per 100 000 live births [7]. EmOC coverage is poor (around 15%) [5]. On the other hand, according to United Nations indicators, there are enough referral centres available and equipped with functional operating rooms. However, the quality of care in the referral centres is inadequate, as evidenced by high case-fatality rates (above 1%) [5].

The concept and techniques of continuous quality improvement offer a variety of strategies to improve the performance of health professionals [8]. These approaches relate to complex interventions in which health professionals are directly involved in analysing and modifying care processes to improve their performance and the health outcomes of their patients. Among these interventions, audit methods may be effective to achieve and maintain high-quality performance of the health workers in low-resource settings [9]. A meta-analysis on audit and feedback approaches that reviewed 47 randomized controlled trials with more than 3500 clinicians showed that this technique may be effective in improving medical practices. The baseline compliance with recommended practice (prior to the intervention) and the intensity of audit and feedback are major factors influencing the effectiveness of this technique [10].

In resource-poor settings, the facility-based maternal death review (MDR) is one of the most-documented audit methods [11-18]. A maternal death review is defined as a "qualitative, in-depth investigation of the causes and circumstances surrounding maternal deaths occurring at health facilities" [19].

When the maternal mortality rate is particularly high, this method helps professionals identify avoidable factors behind deaths, related either to delays in care-seeking or substandard provision of care. Mechanisms to improve care are sought and possible actions are proposed, implemented and monitored. Improvements are also brought about by promoting teamwork and increasing the skills, motivation and accountability of health workers [14].

Observational studies of health facilities in developing countries evaluating MDRs have shown reductions of up to 50% in maternal mortality [14-16]. However, many facility-based MDRs are not published because they are conducted as part of ongoing clinical practice, and so information on the adaptations and difficulties in implementation are not easily obtainable [20]. Each clinical environment presents organizational, professional and cultural particularities that may influence the feasibility and the acceptance of MDR.

The Ministry of Health in Senegal initiated MDR in 2004 in five pilot hospitals with the collaboration of researchers of the University of Montreal. This initiative was the first step of a national programme that aims to scale up MDR in all referral health facilities that offer emergency obstetric care in Senegal. This study's premise is that strategies to implement MDR successfully and reduce maternal mortality should take into account the perceptions of health workers in different contexts in order to identify different factors influencing MDR implementation. Consequently, we carried out an exploratory study to investigate professionals' perceptions of the audit approach, and to identify barriers to and facilitators of its implementation.

## Methods

This study was carried out in five reference hospitals in Senegal with different characteristics (Table 1). The five hospitals were purposely selected to include facilities in Dakar, the capital of Senegal, as well as other areas. They were also selected to include primary-level referral hospitals (district) and more specialized (regional and/or teaching) hospitals.

**Table 1:** Characteristics of participating hospitals

**Characteristics**	**Hospital A**	**Hospital B**	**Hospital C**	**Hospital D**	**Hospital E**
	Teaching/ tertiary level	District	Regional	Regional	Regional

Localization in Dakar (capital city)	Yes	Yes	No	No	No

No. of maternity beds	120	66	54	86	33

No. of doctors covering maternity	7	2	1	3	1

No. of midwives	41	21	9	9	5

No. of deliveries (2004)	6345	7426	2959	4378	648

Availability of basic services^a^	Yes	Yes	Yes	Yes	Yes

Availability of basic emergency obstetric services^b^	Yes	Yes	Yes	Yes	Yes

Availability of caesarean sections^c^	Yes	Yes	Yes	Yes	Yes

Availability of safe blood^c^	No	No	No	No	No

Availability of adult intensive care unit	Yes	No	Yes	Yes	Yes

Number of maternal deaths (2004)^d^	53	44	31	60	37

Overall rate of maternal lethality/1000^e^	8.3	5.9	10.5	13.7	57.1

^a ^Reliable water supply, sanitation facilities, electricity, generator, refrigerator, telephone

^b^Parenteral antibiotics, parenteral oxytocic drugs, parenteral anticonvulsants for pre-eclampsia and eclampsia, manual removal of placenta, removal of retained products (e.g. vacuum aspiration), assisted vaginal delivery (e.g. vacuum extraction, forceps)

^c ^caesarean section and transfusion can be done in the service 364 days/365, 24 h/24

^d^Source of information: registers of deliveries in the maternity units for year 2004

^e^Number of maternal deaths among women giving birth in the facility during the same period

### Hypotheses

Based on a previous study in a district hospital in Dakar [14], we had the following hypotheses: (1) MDR is generally well accepted by health professionals; (2) local leadership is essential to promote and implement MDR in health facilities; (3) traditional hierarchical relationships within health facilities in Senegal may represent a main factor of MDR implementation.

### Implementing maternal death reviews

MDRs were implemented in the five reference hospitals from May 2004 to June 2005, with external support by the National Department of Reproductive Health (NDRH) of the Ministry of Health in Senegal and a nongovernmental organization (CEFOREP). The MDR method was introduced in three stages.

First, a national workshop was held in May 2004 with the heads of the maternity units to present the methodology of MDR and the related research activities. According to the Tenth International Classification of Diseases, the maternal mortality case definition was agreed upon: "the death of a woman while pregnant or within 42 days of termination of pregnancy, irrespective of the duration and the site of the pregnancy, from any cause related to or aggravated by the pregnancy or its management, but not from accidental or incidental causes." A standardized data collection form to collect information about maternal mortality cases and a framework for analysing case management was developed with audit tools from previous studies carried out in Senegal [21,22].

Secondly, audit meeting guidelines were prepared and core audit teams from each hospital, including managers, were trained on-site to identify and analyse maternal mortality cases during September-December 2004.

Thirdly, this preparatory phase was followed by a six-month pilot-testing period of the audit approach in each hospital (from January to June 2005). One member of the NDRH and one member of the CEFOREP visited the maternity units to supervise the audit activities. Findings, audit process, and objectives were reviewed during these visits, with periodic adjustments in methods to better implement the MDR in the various settings.

### Maternal death review method

**Figure 1:**
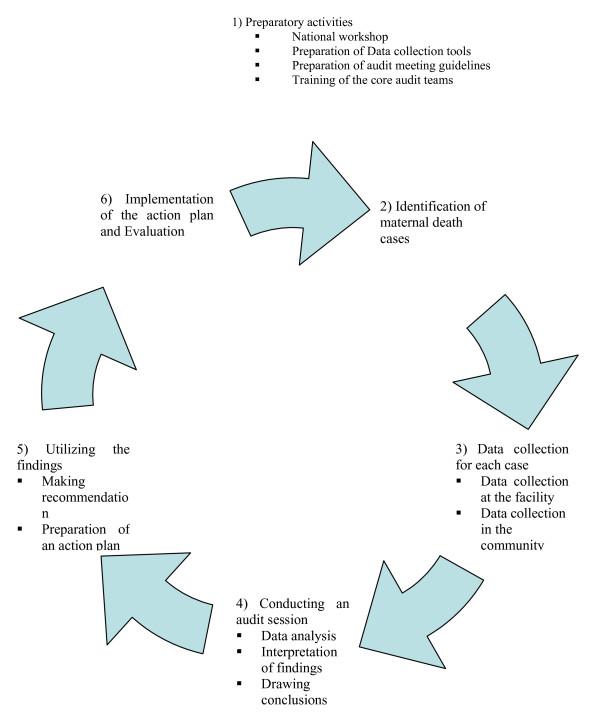
**Steps in the audit process**.

We referred to the method proposed by WHO, presented in detail, in the guide entitled: *Beyond the number: Reviewing maternal deaths and complications to make pregnancy safer *[19]. We defined in advance prerequisites to conduct a facility-based MDR: select data collectors; establish a multidisciplinary audit committee including doctors, midwives, nurses and managers; obtain support and clearance from local health authorities; check for existing record or data systems (registers and medical charts); and check for available protocols for managing major obstetric complications. Practical steps of the audit process are presented in Figure 1.

To monitor the audit process, we asked the professionals to use the two following standard forms: first, the data collection form completed by the data collector for each case of maternal death. This form includes information on maternal characteristics, prenatal care, itinerary before arriving at the hospital, labour and delivery, diagnosed complications and management of the complications. This information was extracted from hospital registers, available medical records and interviews with health workers and members of the family. The second form was the audit report form completed by a member of the audit committee when the case of maternal death had been reviewed. This form includes the conclusions of the committee: the cause of death, factors that contributed to the death, recommendations and the action plan for the immediate future.

### Data sources and collection

The study period started in May 2004, at the beginning of the preparatory phase for the audits, and finished in July 2005, at the end of the six-month pilot-testing period of MDR. Professionals' perceptions were evaluated by means of focus-group discussions; participant observations of audit meetings; audit documents (data collection forms, audit report forms, minutes and lists of attendance at meetings); and interviews with staff of the maternity unit (Table 2).

**Table 2:** Data sources and collection

**Data source**	**Hospital A**	**Hospital B**	**Hospital C**	**Hospital D**	**Hospital E**
Focus group discussion with health personnel	1	1	-	1	-

Participant observations of the audit meetings	1	1	2	2	2

Data collection form (maternal death)^a^	14	27	18	23	23

Audit report form^b^	6	14	13	13	23

Semistructured questionnaire	27	12	8	11	8

In-depth interview with the data collector	1	1	1	1	1

In-depth interview with the head of the maternity unit	-	1	1	1	1

a The data collector (a staff member of the health facility) completes a standard form for each case of maternal death that includes information on maternal characteristics, prenatal care, itinerary before arriving at the hospital, labour and delivery, diagnosed complications and management of the complications. This information is extracted from the hospital registers, from available medical records and from interviews with health workers and members of the family.

b A member of the audit committee completes a standard form when the case of maternal death has been reviewed. This form includes the conclusions of the committee: primary cause of death, factors that contributed to the death, recommendations and action plan for the following weeks.

Three focus groups were conducted by the research team in three hospitals at the beginning of the preparatory phase (May 2004), with four to six participants (doctors and midwives), according to the hospital. Focus groups were separated across hospitals and mixed across professional groups; the discussion lasted approximately two hours and focused on three main themes: determinants of maternal mortality and advantages and disadvantages of maternal deaths reviews.

Eight participant observations were conducted by the research team in five hospitals during the six-month pilot-testing period of the audit process (from January to June 2005). Prior to the visit of the research team, the heads of the maternity units were asked to prepare an audit meeting that would take place on the day of the visit. The main tasks of the observers were to take notes, including non-verbal observations, to record and observe the audit meeting. Audit documents of previous meetings were collected by the observers for quantitative analyses.

At the end of the six-month pilot-testing period of the audits, interviews with staff were conducted in July 2005 by a qualified professional (midwife) who was trained in using the questionnaire. The health authorities provided the research team with lists of the health professionals in each facility. The areas of focus defined for interviews were: sociodemographic characteristics of the health worker; professional qualifications; length of service in the hospital; perception of maternal mortality in the country and in the hospital specifically; participation in training sessions, in the data collection for maternal deaths and in audit sessions; existence of feedback; and perception of barriers to and facilitators of MDRs implementation.

Among the 121 listed professionals of the maternity units, we interviewed those personnel who were currently on staff when the researchers visited the health facility (between two and four days in each centre). Sixty-six (54%) individuals were interviewed: 15 gynaecologists-obstetricians, six other medical practitioners (paediatrician, anaesthesiologist, biologist), 31 midwives, 11 paramedics, three other hospital staff members. After the information sheet was explained, written consent was obtained from participants.

Since the majority of the personnel we interviewed had never participated in the audit meetings, interviews were conducted in the following manner: respondents were asked to describe their perceptions about maternal mortality in their country and in their hospital specifically, barriers and challenges encountered when implementing MDRs and factors and interventions they believed important to facilitating and supporting the audit approach in their hospital. Data collectors (5/5) and the heads of the maternity units (4/5) took part in in-depth interviews that further defined their own specific tasks in implementing MDR.

Focus group discussions, participant observations, semistructured questionnaires and in-depth interviews were conducted in French or in Wolof. At the participants' request, to ensure confidentiality the sessions were not audiotaped. Researchers reconstructed detailed notes immediately after each survey, translated into French if appropriate; together with field-notes, they entered data into a computer by means of Microsoft Office Word software.

### Data analysis

Data were analysed by means of both quantitative and qualitative approaches. Data from the audit documents (data collection form, audit report form, minutes and lists of attendance at audit meetings) were analysed quantitatively to assess the cause of maternal mortality and the recommendations, by means of Epi Info 2000 (Epidemiology Programme Office, Centres for Disease Control, Atlanta, Georgia, United States of America).

Then we used a qualitative approach to investigate professionals' perceptions of the MDR. Analysing the data from one hospital at a time, two researchers independently coded and categorized ideas into broader themes. Focus groups, participant observations, audit documents and individual interviews were separately analysed. Once all documents, questionnaires and detailed notes were analysed, results were reviewed by a third researcher to describe findings that applied to the study as a whole. As hypotheses were generated, the authors sought confirmation by returning to the detailed notes to find evidence to refute or support these.

## Results

### Results of maternal death reviews performed by heath professionals

During the six-month pilot-testing period, 105 data collection forms were completed – one for each registered maternal death in the five hospitals – and 69 (66%) were audited by the five local committees, leading to 69 corresponding audit report forms, including 78 recommendations. The number of participants attending the audit meetings varied from three to eight (including managers but not systematically); one to four cases were reviewed during those meetings.

The cause of death was assessed by the audit committee in 84% of the cases. The main causes of death found by the audit committees were: haemorrhage, pre-eclampsia/eclampsia and uterine rupture. Some 48% of deaths were considered avoidable according to national standards of care; 25% were considered as probably avoidable. The most frequent recommendations were to do as follows: (1) improve initial management of critical patients at admission time; (2) improve the availability of blood for transfusion; (3) improve patient monitoring during the postpartum period.

### Barriers to and facilitators of MDR implementation

The qualitative analysis of the data sources led to the identification of various barriers to (Table 3) and facilitators of (Table 4) the implementation of maternal death reviews. Barriers that were most frequently mentioned by interviewed personnel were: (1) poor quality of information in medical files; (2) lack of involvement of the head of department in the audit meetings; (3) lack of feedback to the staff who did not attend the audit meetings. Facilitators most frequently mentioned were: (1) high level of professional qualifications or experience of the data collector; (2) involvement of the head of the maternity unit, acting as a moderator during the audit meetings.

**Table 3:** Identified barriers to the implementation of maternal death reviews

**Topics**
Factors influencing the identification of maternal death cases:

• Death occurring during the transportation of the woman to hospital or shortly after admission

• Death occurring outside the maternity unit (i.e. in the intensive care unit)

Factors influencing the data collection:

• Poor quality of information in medical files*

• Data collection divided between numerous workers

• Non-permanent collector in a health structure (medical student, resident)

• Non-motivated collector

• Inaccurate address in the medical files, preventing community inquiry

Factors influencing the audit meetings:

• Head of department not involved in the audit meetings*

• Poor quality of the collected information

• Collector is not invited to the audit meetings

• Employees made to feel guilty after audit meetings

Factors influencing the use of the findings:

• Lack of feedback to the staff who did not attend the audit meetings*

• Settings where most of deaths occur because of poor access

*Barriers that were the most frequently mentioned by the interviewed personnel

**Table 4:** Identified facilitators to the implementation of maternal death reviews

**Topics**
Factors influencing the identification of maternal death cases:

• Daily identification of cases

• Consulting many sources of data (hospital registers)

• Computerizing hospital registers

Factors influencing the data collection:

• High level of professional qualifications or experience of the data collector*

• Incentives for the data collector

• Quality of the collector's training

• Interviewing the family members briefly before the exposure of the body

Factors influencing the audit meetings:

• Involved head of department, acting as a moderator during the meeting*

• When possible, information from the community

• Short delay between the death and the audit meeting

• Multidisciplinary meetings

Factors influencing the use of the findings:

• Feedback to the managers and all the staff of the maternity unit

• Involvement of the hospital officials

• Involvement of the community representatives

*Facilitators most frequently mentioned by the interviewed personnel

According to the health professionals interviewed, the perinatal information system in the hospitals was, in general, not suitable to allow an extensive identification of all the maternal deaths occurring in the hospitals. A midwife said:

"Many women die on their way to the hospital or during their admission to the facility: these deaths are not noted in the maternity department records."

The lack of communication between different units in a given health facility was another barrier to the identification of maternal deaths occurring outside the maternity unit (for instance, in the general surgery unit or the intensive care unit). However, in certain health facilities, the designated data collector attended daily staff meetings to get information about maternal deaths that had occurred the day before and completed the registers when necessary. Some collectors even consulted admission registers or registers at the morgue to identify women who had died on their way to the hospital or during admission. In two of the participating hospitals, registers or medicals files were computerized, which greatly facilitated the data collector's task of identifying maternal deaths.

The data collectors of all five hospitals deplored the poor quality of the information in the medical files and said it was difficult to extract information on the itinerary of the woman before arriving in their health facility and the management of the patient after her admission in the maternity unit:

"Doctors sometimes did their diagnosis orally and noted nothing in the medical files, or patients arrived in such a serious state that there was no time to fill the medical files...."

At times, when community enquiry was necessary and possible because of the proximity of the home, the address provided in the medical files was inaccurate and so the family of the deceased and her circle were not located. Professionals recognized that it was easier for a person with experience and a high position in the hospital's hierarchy than a junior person to conduct interviews with other members of the staff, especially when enquiring about maternal death cases.

Even though audit meetings were regularly organized in all five hospitals, the recommendations provided by our team about the audit process were not respected everywhere. In particular, data collectors were not systematically invited to audit meetings, and at times, the number of participants at these meetings was very low (usually doctors).

In some of the hospitals, the weight of traditional hierarchical relations between doctors and other categories of personnel within the maternity unit was a barrier to establishing a multidisciplinary audit committee. This situation was one of the reasons why the personnel weren't motivated to collect information on maternal deaths or to implement the audit committee's recommendations. Some of the interviewed professionals complained of a lack of communication between the audit committee and the staff:

"I was not invited to participate in the audit meetings and I was never informed of the conclusions. It was disappointing..." (a surgical assistant at one hospital)

One head of the maternity unit who was interviewed believed that only doctors could conduct an audit session: "... because midwives and nurses were not qualified enough to give solutions and correct doctors..."

In certain cases, the midwives who participated in audit meetings considered the situation destabilizing or even threatening:

"I felt guilty, even threatened by a penalty. The head of department said that the woman was killed when he talks about what happen in the unit when the patient died."

Conversely, in most of the hospitals where the head of the maternity unit did not attend the audit meetings, the employees were not motivated to participate in the process, because they felt that their recommendations would not be implemented.

### Suggestions from the interviewed personnel to improve implementation

The interviewed professionals made several suggestions to improve the audit meetings, particularly the participation at these meetings:

• First, invite all employees to the meetings, or at least one representative of each category of professionals.

• Providing food at the meetings would also motivate people to participate and would create a more convivial atmosphere during these sessions.

• Furthermore, organizing the meeting early after the death occurred, and presenting the information obtained from the community when a visit to the family or relatives was made, would permit a deeper analysis and stimulate more discussion about the case.

• According to the interviews, the head of department must always be present at these meetings and play a moderating role.

• The conclusions of the audit meetings should be transmitted to the hospital's administration and regional authorities.

• Feedback to the health workers should be formalized as a memo posted in the staff room; the information in the memo should be anonymous.

## Discussion

### MDR is generally well accepted by health professionals

Health professionals and service administrators were receptive and adhered relatively well to the process and the results of the audits, as evidenced by the number of maternal death cases audited, and the relevance of the recommendations drawn by the local audit committees. The focus groups conducted during the preparatory phase clearly had value as orientation/training and should be recommended when involving a new facility.

The results of the maternal death audits performed by health professionals in the five pilot hospitals are consistent with the cause and recommendations that are presented in the scientific literature [11-14,18,21,22]. The main causes of death identified by other researchers in sub-Saharan Africa are haemorrhage, pre-eclampsia/eclampsia and obstructed labour. Sub-standard care was identified in 60% to 80% of cases. The main factors to improve in order to prevent maternal deaths include the quality of care at admission (specifically for critically ill patients) and in the postpartum period (for an appropriate management of complications). Improving the availability of blood for transfusion was identified as a priority by other authors in sub-Saharan Africa [14,15].

### Leadership is a strong facilitator of MDR implementation

Results of this exploratory study suggest that the implementation of MDR in low-resource settings is strongly influenced by the quality of the perinatal information system, the professional qualifications or experience of the data collector and the leadership of the head of the maternity unit. In the hospitals where local leadership was inadequate, health care professionals described the situation as destabilizing or even threatening and the feedback of audit results and recommendations was ineffective. In the hospitals where the head of the maternity unit was involved in the audit process, the audit approach was generally accepted by health professionals.

The choice of the person to assume the role of data collector seemed to have greatly influenced the implementation of the MDR in the health facilities. We realized that data collection was generally less efficient when it was divided among several people, or when the task was assigned to a student, a resident or a permanent employee who had little motivation for this work. Generally, the data collection procedure encountered no major problems when the task was assigned to a professional who had experience and to whom this kind of work was part of his or her field of competence. An example of such an individual would be a midwife who was also responsible for the coordination of services in the maternity unit or at the district or regional level.

Some financial motivation and a good initial training usually enabled the participants to reach an adequate level of collaboration and adhesion to the audit guidelines [14,19,20,23]. Moreover, the data collection in the maternity wards can be improved as a part of quality assurance programmes. Information routinely collected by health professionals (medical files and registers) should be used to develop a valid information system that would help health workers and managers monitor maternal and prenatal health in real time. Standardized medical files or partograms are needed to monitor at regional or national level. Regular checking of data by trained supervisors is essential.

### Traditional hierarchical relationships may be a facilitator under specific conditions

The hierarchy within a given community has a great impact on social relationships in Senegal, and particularly among health care professionals [24]. Few authors have stressed the important role of the head of the maternity unit in reducing hierarchical boundaries, promoting a multidisciplinary approach and increasing staff acceptance of the MDR. In West-African countries, because of the professional hierarchy and the organization of health facilities, the head of the maternity unit is a key actor for the implementation of the MDR. However, his or her capacity to demonstrate his or her ability to engage proactively in the audit process and dialogue with the staff to advance the common goals of the MDR depends on the following key aptitudes: (1) knowledge of evidence-based practice for the main obstetric complications; (2) an understanding of non-medical reasons for maternal death (social, economic, cultural and legal dimensions of maternal mortality); and (3) a mastery of the audit approach [14,25].

Another key actor in MDR implementation is the data collector for maternal mortality cases. Data collectors interviewed in this study confirmed that performing the interview with the personnel and the family of the deceased woman is difficult. A person with experience and a senior position in the hospital's hierarchy, who is well respected in the community, may collect the information better than a younger employee or a subordinate. However, the person conducting this kind of interview should be very tactful and sensitive [23]. He or she should be aware of the concept of the three delays that limit the access of the women to health care services [26].

The WHO *Beyond the numbers *manual recommends involving hospitals and service managers in the MDRs in order for them to understand well the issues and to work on recommendations with the other professionals [19]. The participation of managers in the audit session is then essential to built teamwork, to facilitate the review of maternal death in a constructive way and to plan appropriate and realistic actions to prevent other maternal deaths.

## Conclusion

The results of this study in Senegal suggest that the maternal death audit approach is generally accepted by health professionals when the information collected for the audit is appropriate and local leadership is strong enough to promote non-threatening and multidisciplinary audit meetings. Since we selected different hospitals with various characteristics, these results could be generalized to other health facilities in Senegal and in other countries with similar contexts to West Africa. We recommend for future implementation of this method the following principles:

1. prior enhancement of the perinatal information system. The hospital's administration must help the health workers archive different data sources to be able to gain access to them easily, or even record selected information in a computerized system. Medical files must be classified and organized in a specific room, which should be locked at all times to preserve confidentiality.

2. appropriate choice and training of the data collector. The data collector should be an experienced professional who has a senior position in the hospital hierarchy. The objectives of initial training are: to improve competence in interviewing staff and family members after a maternal death; to carry out the door-to-door approach to describe the patient's management within the health system; and to synthesize the information for the audit committee.

3. appropriate training of the head of the maternity unit to increase his or her leadership. The objectives of this training are to improve his or her knowledge of best practices and of the social, economic, cultural and legal factors that hinder women's access to essential services, as well as audit procedures and teaching techniques to adults.

4. appropriate training of the entire team in the audit process to facilitate its implementation.

## Competing interests

The authors declare that they have no competing interests.

## Authors' contributions

AD participated in developing the project and is responsible for the scientific aspects of the research and all its components. PF participated in developing the project. AD and PF obtained the funding for the project. CT was responsible for the coordination of the research activities in Senegal. AD wrote the first version of the manuscript and coordinated its development. All authors read and approved the final manuscript
